# Frequent mechanical stress suppresses proliferation of mesenchymal stem cells from human bone marrow without loss of multipotency

**DOI:** 10.1038/srep24264

**Published:** 2016-04-15

**Authors:** Viktoria Frank, Stefan Kaufmann, Rebecca Wright, Patrick Horn, Hiroshi Y. Yoshikawa, Patrick Wuchter, Jeppe Madsen, Andrew L. Lewis, Steven P. Armes, Anthony D. Ho, Motomu Tanaka

**Affiliations:** 1 Physical Chemistry of Biosystems, Institute of Physical Chemistry, University of Heidelberg, 69120 Heidelberg, Germany; 2 Department of Medicine V, University of Heidelberg, 69120 Heidelberg, Germany; 3 Department of Chemistry, Saitama University, 338-8570 Saitama, Japan; 4 Institute for Integrated Cell-Material Sciences (WPI iCeMS), Kyoto University, 606-8501 Kyoto, Japan; 5 Department of Chemistry, Dainton Building, University of Sheffield, Brook Hill, Sheffield, South Yorkshire, S3 7HF, United Kingdom; 6 Biocompatibles UK Ltd, Chapman House, Farnham Business Park, Weydon Lane, Farnham, Surrey GU9 8 QL, United Kingdom

## Abstract

Mounting evidence indicated that human mesenchymal stem cells (hMSCs) are responsive not only to biochemical but also to physical cues, such as substrate topography and stiffness. To simulate the dynamic structures of extracellular environments of the marrow *in vivo*, we designed a novel surrogate substrate for marrow derived hMSCs based on physically cross-linked hydrogels whose elasticity can be adopted dynamically by chemical stimuli. Under frequent mechanical stress, hMSCs grown on our hydrogel substrates maintain the expression of STRO-1 over 20 d, irrespective of the substrate elasticity. On exposure to the corresponding induction media, these cultured hMSCs can undergo adipogenesis and osteogenesis without requiring cell transfer onto other substrates. Moreover, we demonstrated that our surrogate substrate suppresses the proliferation of hMSCs by up to 90% without any loss of multiple lineage potential by changing the substrate elasticity every 2nd days. Such “dynamic *in vitro* niche” can be used not only for a better understanding of the role of dynamic mechanical stresses on the fate of hMSCs but also for the synchronized differentiation of adult stem cells to a specific lineage.

Human mesenchymal stem cells (hMSCs) can undergo both self-renewal and differentiation into multiple lineages, which makes them highly attractive for applications in regenerative medicine and tissue engineering^1^. Recent experimental evidence indicates that mechanical properties of the microenvironment, as well as biochemical stimuli, determine the long-term fate of stem and progenitor cells^2^,^3^. Cells can actively sense and respond to the mechanical properties (elasticity) of the surrounding extracellular environments by the clustering of integrin receptors. This leads to the formation of focal adhesions that facilitate the downstream cascades of intracellular signaling pathways. Such adhesion-induced signaling pathways, called as outside-in signaling, trigger the generation of forces by the contracting actin-myosin (actomyosin) complexes^4^. The resistance of substrates against the applied traction force controls signaling molecules, such as talin-vinculin complexes, which mediate the connection between integrin clusters and actomyosin complexes^5^. On the other hand, the stimulation of actomyosin contraction can also lead to the conformational change in the cytoplasmic domains of integrin molecules, which increases the binding affinity of the extracelluar domain (inside-out signaling)^6^.

To date, various extracellular matrix (ECM) models based on chemically cross-linked hydrogels have been developed in order to understand how such a positive feedback in mechano-sensing regulates the fate of stem and progenitor cells. Fine tuning of the cross-linker concentration and the reaction time^7^,^8^ enables one to control the elastic modulus of a given gel substrate. Such “*ex situ*” regulation of the mechanical microenvironment of cells provides important insight into the vital role of elasticity compliance in optimizing the cell morphology^9^,^10^,^11^, as well as regulating the migratory behavior^12^,^13^,^14^, and the differentiation fate of human mesenchymal stem cells (hMSCs)^15^,^16^. Engler *et al*.^15^ functionalized the surface of chemically cross-linked polyacrylamide gels with type I collagen, and demonstrated that the multiple lineage differentiation of hMSCs can be controlled via the substrate elasticity. Winer *et al*.^17^ reported that hMSCs seeded on very soft polyacrylamide gels (*E* ~ 250 Pa) with fibronectin coatings sustained their multi-lineage differentiation capacity and suppressed cell proliferation. Zemel *et al*.^18^ calculated the nematic order parameters of actomyosin complexes by staining both actin and non-muscle myosin II (NMMII) for hMSCs on polyacrylamide gels, and found that the maximum order parameter correlated directly with the optimal substrate elasticity for muscular tissues. However, these *ex situ* approaches have a fundamental problem to model dynamic mechanical microenvironments of stem cells^19^. As suggested by *in vivo* studies, cells are highly sensitive to dynamic changes in their mechanical environment both during their development and when subjected to disease. One biologically relevant example is the significant influence of ECM density on the transition of cancer cells from a protease-dependent movement to an amoeboid movement^20^. To provide stem cells with dynamically tunable mechanical environments, stimulus-responsive polymers have been the focus of increasing attention for biomedical applications^21^,^22^,^23^,^24^,^25^,^26^,^27^. For example, Okano and co-workers designed substrates based on thermo-responsive poly(N-isopropylacrylamide)-based hydrogels for the formation of two-dimensional cell sheets, which can be readily detached from culture dishes below the low critical solution temperature for transplantation^28^,^29^. More recently, Yang *et al*.^30^ reported the control of hMSC on poly(ethylene glycol) hydrogel, whose elastic modulus can be lowered from 10 kPa–2 kPa by UV illumination.

In our previous study, we utilized a copolymer hydrogel that exhibits a substantial change in mechanical properties in response to subtle changes in solution pH^31^,^32^. This copolymer gel comprises a highly biocompatible, pH-responsive, physically cross-linked ABA-type triblock copolymer [A = poly(2-(diisopropylamino)ethyl-methacrylate), (PDPA) and B = poly(2-(methacryloyloxy)ethyl-phosphorylcholine), (PMPC)] (Fig. 1). The central PMPC block (for which the mean degree of polymerization *n*, is 250) confers excellent biocompatibility^33^,^34^,^35^,^36^, while the mean degree of ionization of the two outer PDPA blocks (for which *n* = 50 for each block) changes significantly at around neutral pH^37^. We reported that the Young’s modulus, *E*, can be modulated reversibly from 1.4 kPa–40 kPa by adjusting the pH between 7.0 and 8.0 32,38, this is an ideal range since it encompasses the gel stiffness of various tissues. The kinetics of elasticity modulation is determined by that of pH titration, which is in order of minutes^32^. Mouse myoblast cells (C2C12) repeatedly undergo transition between contractile and hemi-spherical morphologies, demonstrating that such pH-responsive hydrogels enable appropriate dynamic mechanical cues, which are applied to cells^31^,^32^. In this study, we utilized PDPA-PMPC-PDPA copolymer gels as a novel tool to manipulate the stiffness of environmental substrates, hence providing dynamic mechanical cues to the hMSCs as surrogate niche models, with subsequent modification of the stem cell fate. By avoiding covalent coupling of adhesion motifs (such as type I collagen)^39^,^40^, we minimize the adhesion-induced interference with the fate of hMSCs and address the following important questions: Can PDPA-PMPC-PDPA gels sustain the multiple lineage potential of hMSCs? How do hMSCs sense the dynamic stiffening/softening of gel substrates? Is it possible to influence the fate of hMSCs by applying periodic mechanical stresses?

## Results

### Morphological Response of hMSCs to Surrogate Elasticity (*ex situ*)

**Figure 2:**
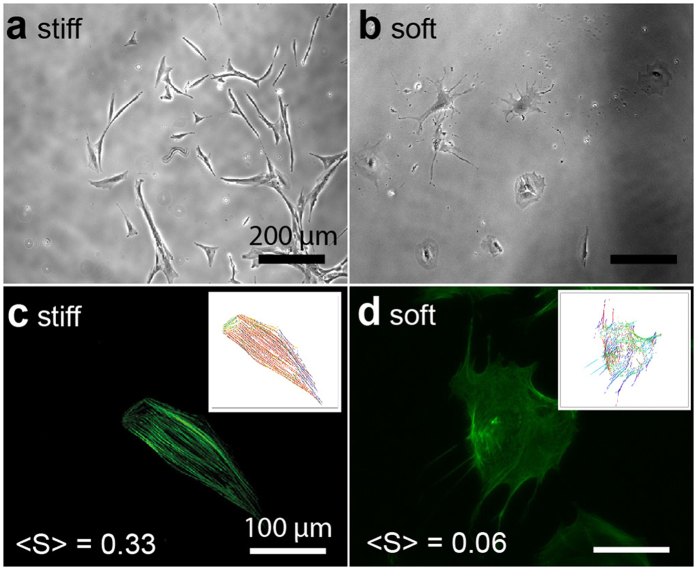
Morphological response of bone marrow-derived, human mesenchymal stem cells (hMSCs) on PDPA_50_-PMPC_250_-PDPA50 copolymer gels. Phase contrast images of hMSCs at *t* = 10 d on (**a**) stiff (*E* = 40 kPa) and (**b**) soft (*E* = 2 kPa) substrates. Fluorescence images of phalloidin-labeled actin (green) at *t* = 10 d on (**c**) stiff (*E* = 40 kPa) and (**d**) soft (*E* = 2 kPa) substrates. The order parameters <*S*> of actin cytoskeletons can be extracted from the pixel orientation maps calculated from the original images are presented as insets.

Figure 2a,b represent the phase contrast images obtained for hMSCs placed on “stiff (pH 8.0, *E* = 40 kPa)” and “soft (pH 7.2, *E* = 2 kPa)” PDPA-PMPC-PDPA copolymer gels, respectively. hMSCs became anisotropically spread on stiff gels after 10 d. In fact, when the actin cytoskeleton was labeled with phalloidin-Alexa Fluor 488 after fixation, a pronounced stress fiber formation was observed (Fig. 2c). In contrast, hMSCs spreading on the soft copolymer gel was much more isotropic, and no distinct order of stress fibers could be identified (Fig. 2d).

The difference in cell morphology can be further assessed by calculating the nematic order parameter of actin cytoskeletons, 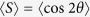 with the aid of an elongated Laplace function of a Gaussian filter to obtain maximum response^18^,^31^,^41^. As presented in the insets shown in Fig. 2c,d, hMSCs on the stiff copolymer gel exhibit a much higher order parameter, 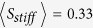, than on the soft copolymer gel, 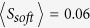. Previous reports demonstrated that hMSCs adapt their shape and cytoskeletal order in response to the elasticity of the underlying substrate, and hence commit to the specific lineage via such mechanical cues^15^,^17^. Thus, the observation of a clear difference in cell morphology (Fig. 2) led to the next question. Do hMSCs cultured on stiff and soft substrates differentiate into the terminal lineage? In other words: does the difference in morphology coincide with the lineage commitment?

### Maintenance of hMSC STRO-1 Expression is Independent from Substrate Elasticity

**Figure 3:**
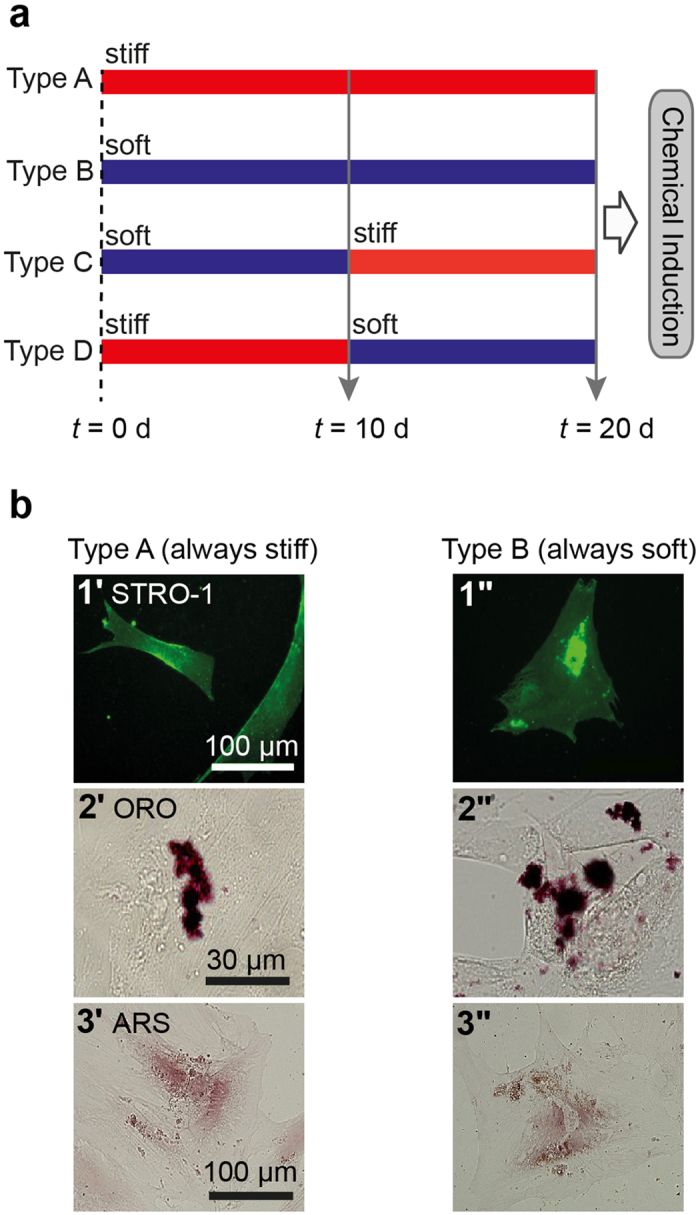
hMSCs sustains multipotency after 20 d independent from substrate stiffness. (**a**) Flow of experiments: Type A; substrate elasticity was always kept “stiff” (*E* = 40 kPa) for 20 d, Type B; substrate elasticity was always kept “soft” (*E* = 2 kPa) for 20 d, Type C; substrate elasticity was switched from “soft” to “stiff” at *t* = 10 d, Type D; substrate elasticity was switched from “stiff” to “soft” at *t* = 10 d. (**b**) Fluorescence images of hMSCs always cultured on stiff substrates (Type A) and soft substrates (Type B). Labels: (**b1**) anti-STRO-1, (**b2**) Oil Red O (ORO), and (**b3**) Alizarin Red S (ARS).

Figure 3a represents the flow of 4 × series of experiments to unravel the influence of substrate elasticity on the fate of hMSCs. hMSCs categorized as Type A were cultured always on substrates whose elasticity was always kept “stiff” (*E* = 40 kPa), while Type B hMSCs were cultured always on soft substrates (*E* = 2 kPa). Type C hMSCs were first cultured on soft substrates (*E* = 2 kPa) for 10 d, and the elasticity was switched to *E* = 40 kPa (stiff). In case of Type D hMSCs, the substrate elasticity was switched from “stiff” to “soft” at *t* = 10 d. Prior to the experiments, we confirmed that pH modulation did not interfere with the viability (SI Fig. S1) and morphology (SI Fig. S3) of hMSCs.

Figure 3b1 represents the immunofluorescence images of Type A hMSCs (3b1’) and Type B hMSCs (3b1”) at *t* = 20 d, labeled with antibody to a mesenchymal stem cell marker STRO-1^42^,^43^. A substantial majority (~90%) of the hMSCs out of approx. 40 cells cultured on both stiff and soft substrates exhibited positive STRO-1 signals. This is in contrast to the same hMSCs cultured on plastic dishes, where we found the fraction of cells exhibiting positive STRO-1 signals decreased to 15–20% already within 10 d (SI Fig. S6), which agrees well with the previous account^43^. To further verify the multiple lineage potential of hMSCs cultured on PDPA-PMPC-PDPA copolymer gels, the media were exchanged to induction media for osteogenesis and adipogenesis at *t* = 20 d. After hMSCs were further cultured for 28 d, hMSCs were subjected to the labeling with Oil Red O (ORO) for adipogenesis (Fig. 3b2) and Alizarin Red S (ARS) for osteogenesis (Fig. 3b3), respectively. Both Type A hMSCs and Type B hMSCs showed positive signals for ORO and ARS, suggesting hMSCs retain multiple lineage capability irrespective of the substrate stiffness. The Type C and Type D hMSCs showed the same tendency for the anti STRO-1 labeling, adipogenesis and osteogenesis (SI Fig. S7). It should be noted that the size of lipid droplets in hMSCs cultured on hydrogel substrates (Fig. 3b2) seemed smaller, and the number of droplets in cells seemed much less compared to those on confluent hMSCs cultured on plastic dishes (SI Fig. S4). Different from conventional culture conditions of hMSCs, we modulated pH using HEPES-buffered medium and seeded hMSCs at a density (10^4 ^cell/cm^2^). The former is essential because pH modulation between 7.2 and 8.0 is not possible by using commonly used hydrogen carbonate buffer. The latter condition was selected because the main focus of the study is to elucidate the mechanical response of individual hMSCs. In order to understand if such a difference potentially be attributed to the influence of culture media (hydrogen carbonate buffer vs. HEPES buffer), we first compared hMSCs cultured on plastic dishes in two different media. Lipid droplets in hMSCs cultured in hydrogen carbonate-buffered medium (SI Fig. S4a) were much more pronounced compared to those in hMSCs cultured in HEPES-buffered medium (SI Fig. S4b), suggesting that the use of HEPES caused a poor lipid droplet formation. Moreover, we found that the decrease in cell density resulted in a clear decrease in the lipid droplet formation in both media (SI Fig. S4c,d), which agrees well with the previous report by McBeath *et al*.^44^. Thus, it has been concluded that an apparently poorer lipid droplet formation on gels (Fig. 3b2) was caused by the use of HEPES buffer as well as the low cell density. In case of osteogenetic induction, the formation of calcinated extracellular matrix could be identified by positive ARS signals for hMSC cultured on both stiff and soft hydrogel substrates (Fig. 3b3). Although ARS signals in SI Fig. S5a also seemed weaker compared to the signal intensity from hMSCs cultured on plastic dishes kept in the same induction medium (SI Fig. S5a), a negative control experiment of the same hMSC in growth medium (SI Fig. S5b) confirmed the specificity of the labeling. Based on the above mentioned results, we concluded that hMSCs cultured on PDPA-PMPC-PDPA copolymer gels sustained the capability to undergo both adipogenesis and osteogenesis after *t* = 20 d, independent from the substrate elasticity.

**Figure 4:**
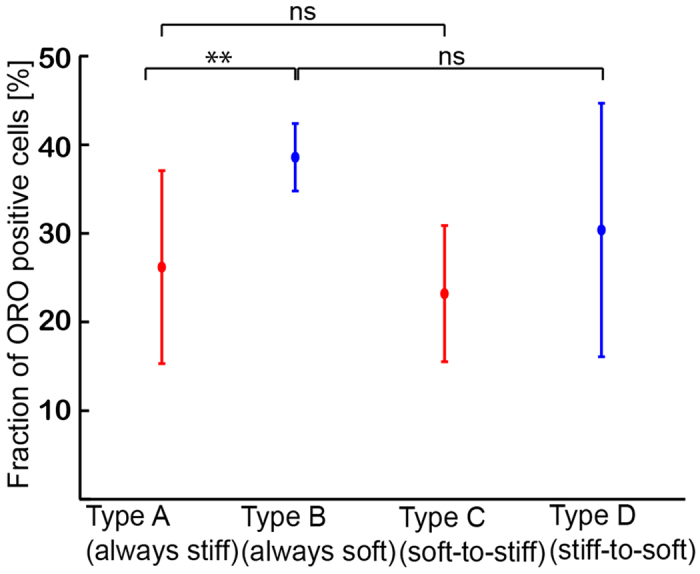
Fraction of ORO positive hMSCs cultured in adipogenic induction medium for 28 d. Prior to induction hMSCs were cultured for 20 days on hydrogel substrates as illustrated in Fig. 3a. Significance levels p < 0.05 by Wilcoxon-test.

The next question we wanted to address if the efficiency of chemical induction is influenced by the mechanical environment. As presented in Fig. 4, 26 ± 11% of Type A hMSC (approx. 50 cells for each condition) exhibited positive ORO signals, while the corresponding value for Type B hMSC was 38 ± 4%. When hMSCs experienced a change in substrate elasticity at *t* = 10 d, the fractions of cells showing positive ORO signals were found to be 24 ± 8% for Type C hMSCs and 30 ± 14% for Type D hMSCs, respectively. Despite of relatively large errors in Fig. 4, the statistical analysis (Wilcoxon test) suggest that the adipogenic induction efficiencies of Type B and Type D hMSCs were distinctly higher compared to those of Type A and Type C hMSCs. This suggests that Type C and Type D can readopt to their new mechanical environments and cancel the mechanical memory of initial substrates. It should be noted that the fraction of hMSCs undergoing osteogenesis could not be quantified in case of osteogenic induction, because ARS identifies calcium compounds of the extracellular matrix but not osteogenic cells. Our finding clearly differs from the previous account^15^, which reported that hMSCs commit to terminal differentiation after just 14 d. Currently, this apparent discrepancy can be attributed to the lack of collagen type I on the surface. In fact, several studies suggested that type I collagen and vitronectin on the surface could bias the MSC lineage towards osteogenesis^39^,^40^,^45^. In contrast, the surface of copolymer gel [Fig f5]substrates was coated by fibronetin physisorbed from the cell culture medium^32^,^31^.

### Reversible Morphological Dynamics of hMSCs to Mechanical Stresses (*in situ*)

**Figure 5 f5:**
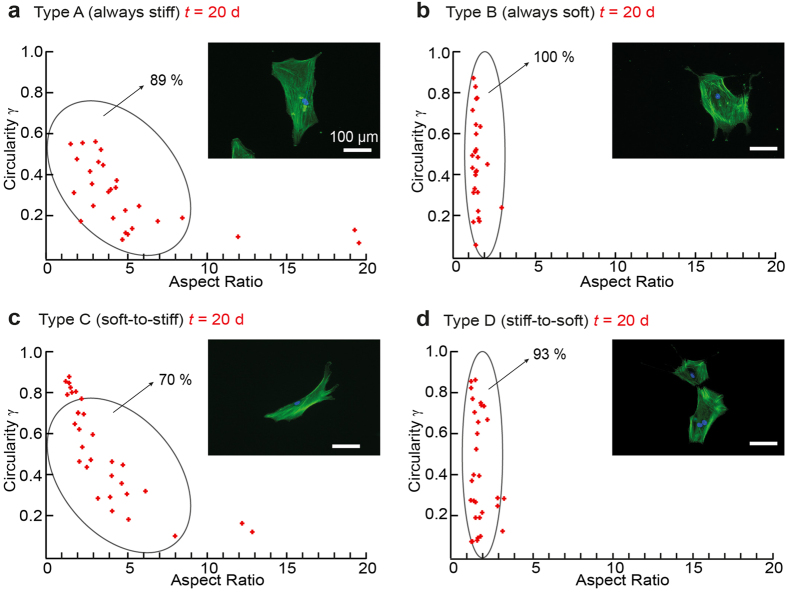
Morphological phenotypes of hMSCs. (**a**–**d**) Plot of circularity *γ* vs. aspect ratio *AR* for Type A–Type D hMSCs at *t* = 20 d. Representative fluorescence images (actin: green, nucleus: blue) are presented in insets. Each data set represents from *n* > 30 cells. Morphological populations were grouped by two ellipses characteristic for Type A hMSCs (89%) and Type B hMSCs (100%). Type A phenotype can represent 70% of Type C hMSCs, while Type B phenotype represents 93% of Type D hMSCs.

The fact that the hMSCs could undergo multiple lineage commitments even after 20 d (Fig. 3b) suggests that the morphology is not the decisive parameter that direct the fate of hMSCs. As presented in Fig. 4, hMSCs were able to adopt their shape in response to the change in substrate elasticity and cancel the mechanical memory of initial environments after 10 d. To identify the characteristic morphological patterns, the morphology of Types A–D hMSCs was categorized by plotting the circularity 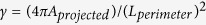 versus aspect ratio *AR*, which is the length/width ratio defined by an ellipsoidal fit at *t* = 20 d (Fig. 5). The *γ*-*AR* map of Type A hMSCs attains a maximum *γ* value of ~0.6 at *AR* = 1–5. This main group (89%) is accompanied with a very broad tail towards larger aspect ratios (*AR* ~ 20), suggesting that the cells were anisotropically stretched. On the other hand, *AR* values observed for Type B hMSCs are concentrated within a narrower range (*AR* = 1–3), showing widely scattered *γ* values. This pattern covering all Type B hMSCs coincides with the isotropic spreading of hMSCs on soft gels, with extended spiky protrusions (filopodia). Once the substrate stiffness is switched from “soft” to “stiff” at *t* = 10 d (Type C), the morphological pattern and the order parameter at *t* = 20 d appear to be very similar to that of Type A cells: the value of actin order parameter for the Type A does not differ significantly from Type C <*S*_A_> = 0.33 ± 0.17 and <*S*_C_> = 0.36 ± 0.12 (SI Fig. S2). In contrast, the morphology of Type D hMSCs (experiencing the change in substrate stiffness from “stiff” to soft” at *t* = 10 d) becomes almost identical to that of Type B, which can also be characterized by a very low order parameter, <*S*_B_> = 0.02 ± 0.18 and <*S*_D_> = 0.18 ± 0.18 (*n* > 9 cells). The values of the actin order parameter for the Type B and Type D are not significantly different (SI Fig. S2). In summary, these results indicate that hMSCs retain their ability to adopt not only the nematic order of actin stress fibers (Fig. S2) as well as their morphological patterns in both directions (“soft to stiff” and “stiff to soft”) after 10 d.

**Figure 6:**
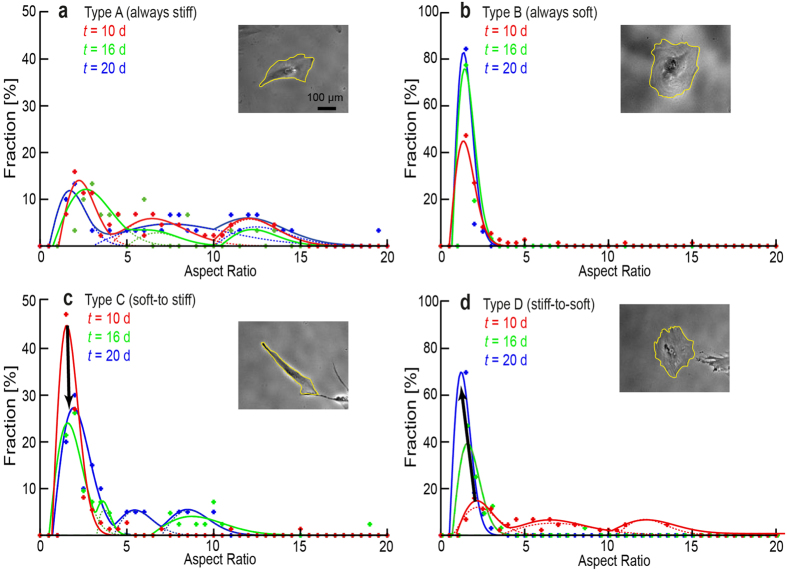
Dynamic morphological response of hMSCs to mechanical stresses over time. (**a**–**d**) Change in aspect ratio (*AR*) of hMSCs vs. time for four types of hMSCs (Type A–Type D). Each histogram was extracted from *n* > 30 cells. Phase contrast images of representative cells for the four types (Type A–D) at *t* = 20 d are presented as insets. The cell contour was highlighted in yellow.

In a subsequent step, dynamic changes in morphological patterns of hMSC induced by the change in substrate stiffness were monitored over time. Figure 6 represents the histograms of aspect ratio *AR* measured at *t* = 10 d (red), 16 d (green), and 20 d (blue) for the four aforementioned four types of hMSCs. Type A hMSCs exhibit three characteristic peaks at *AR* ~ 3, 7, and 12 over time (Pattern A), while Type B hMSCs are characterized by a single peak near *AR* ~ 1 (Pattern B). When hMSCs experience a change in the substrate stiffness from “soft-to-stiff” (Type C), hMSCs undergo a transition from Pattern B (soft) to Pattern A (stiff) over time. On the other hand, *AR* of Type D hMSCs changed from Pattern A to Pattern B. If one generalizes the morphological transition of hMSCs upon an abrupt change in the substrate elasticity as a non-equilibrium relaxation process, the characteristic time constants in both directions were estimated from the change in intensity of the principal peaks with the smallest *AR* to be *τ* = 3–4 d. The hMSC response to such an “elasticity jump” appears to be much slower than that of myoblasts C2C12 (*τ* = 10–30 min)^32^,^31^. The projected area of hMSCs (n = 30 cells, *t* = 10 d), *A*_hMSC_ (soft) = (1.8 ± 0.9) ×10^4 ^μm^2^ and *A*_hMSC_ (stiff) = (1.1 ± 0.7) ×10^4 ^μm^2^, was about two orders of magnitude larger than that of C2C12 32.

This is the first demonstration of the reversible switching of STRO-1 positive hMSC morphology, which offers a unique advantage to understand the mechanism regulating the dynamics of morphological response and remodeling actin cytoskeletons. This is of particular interest, not only for maintaining hMSC multiple lineage potential, but also for optimization of the hMSC-based feeder layers for the expansion of human hematopoietic stem cells (hHSC) *in vitro*^46^. From this context, a recent study indicated that the cytoskeletal protein nestin may play a critical role in the maintenance of stemness of mouse HSC in the bone marrow niche^47^.

### Frequent Mechanical Stresses Suppress Proliferation of hMSCs

**Figure 7:**
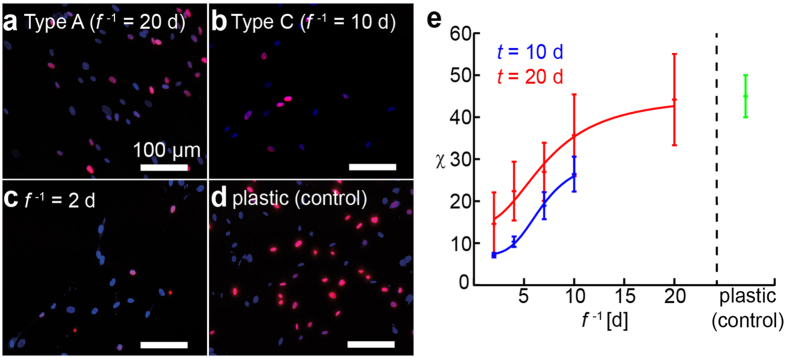
Impact of the frequency of mechanical stress *f*^−1^ on hMSC proliferation. (**a**–**d**) Dual staining with DAPI (blue) and anti-BrdU (magenta) of hMSCs (*t* = 20 d) experiencing: (**a**) no change (Type A), (**b**) *f*^−1^ = 10 d (Type C), (**c**) *f*^−1^ = 2 d. (**d**) hMSCs cultured on plastic dishes (control). (**e**) Fractions of anti-BrdU positive (proliferating) cells *χ* plotted as function of duration of a mechanical step *f*^−1^ at *t* = 10 d (blue) and 20 d (red) exhibiting a non-linear relationship between *χ* and *f*^−1^. Each data point represents mean values ± SD for *n* > 30 cells.

Consideration of the above results naturally leads to the following two questions. Is it possible to influence the fate of hMSCs by applying periodic mechanical stresses? Is there a characteristic frequency that is particularly effective for influencing cell fate? To address these fundamental questions, we examined the incorporation of bromodeoxyuridine (BrdU) into the DNA of hMSCs subjected to mechanical jumps of different frequencies. Figure 7a–d depict fluorescence images of hMSCs that were fixed and stained with 4′,6-diamidino-2-phenylindole (DAPI, blue) and anti-BrdU (magenta) after overnight incubation with BrdU at *t* = 20 d. Comparing the proliferative behavior of hMSCs experiencing no change on a stiff gel substrate (Type A, Fig. 7a), one change at *t* = 10 d (Type C, Fig. 7b), nine changes at *t* = 2, 4, 6, 8, 10, 12, 14, 16 and 18 d (Fig. 7c), and hMSCs cultured on a plastic dish (control, Fig. 7d), it is clear that the fraction of proliferating cells at *t* = 20 d exhibits a dramatic reduction as the frequency of mechanical switching *f*^*−1*^ is increased. As summarized in Fig. 7e, the fraction of proliferating cells *χ* plotted as a function of the duration of one mechanical step (and thus *f*^−1^) at *t* = 10 d (blue) and 20 d (red) shows a non-linear behavior. The resulting *χ* vs. *f*^−1^ relationship can be fitted with an empirical Hill equation^48^:


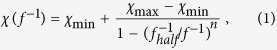


where *χ*_max_ and *χ*_min_ stand for the maximum and minimum values of *χ*, 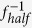 = 6.5 d for the characteristic duration of the transition between proliferative and no-proliferative cells, and *n* ~ 7 for the cooperativity at *t* = 10 d. We found that a frequent mechanical stress applied to hMSCs can suppress proliferation by approximately 90 ± 5% after maintaining hMSCs on copolymer gel substrates for 10 d. Moreover, the suppression of proliferation was still prominent even after 20 d, where we found 85 ± 9% of cells remained non-proliferative.

## Discussion

There have been an increasing number of papers reporting the vital roles of mechanical properties of stem cell niche in directing the fate of stem cells. For example, Katayama *et al*.^19^ reported that the sympathetic nervous system induced the stiffening and flattening of osteoblasts in bone marrow, which regulates the egress of hematopoietic stem cells. Cameron *et al*.^49^ demonstrated that the substrates possessing a high loss modulus (viscosity) can keep the contractility of actin cytoskeleton low. More recently, Zhang *et al*.^42^ reported that the multipotency of hMSC is sustained if the contractility of actomyosin is kept low. However, despite major achievements in the last decade, most of experimental studies have been performed on gel substrates whose elasticity was fixed as a function of the degree of chemical cross-linking. This raised the issue if such *in vitro* niche models are biologically relevant, as cellular micro-environments are known to be highly dynamic. Recently, Guvendiren *et al*.^50^ prepared hydrogels with tunable elasticity by functionalizing hyaluronic acid with methacrylic anhydride (HA-MA). Such gels became dynamically stiffened (from *E* = 3–30 kPa) after injection of dithiothreitol (DTT) by cross-linking the pendent MA groups via radical polymerization under UV irradiation. After functionalization of surfaces with RGD motifs, they found that adipogenesis is favored for later stiffening (*t* > 8 d) while osteogenesis is preferred for earlier stiffening (*t* < 8 d).

Within this context, PDPA-PMPC-PDPA copolymer gels offer a unique advantage over the above mentioned materials owing to its capability of switching the elasticity in a *bidirectional* reversible manner. As the hMSC marker, we utilized the surface marker STRO-1, as Simmons *et al*.^43^ reported that colony forming unit factor (CFU-F) is present exclusively in STRO-1 positive populations. Other authors have confirmed that STRO-1 could be used to sort hMSCs that possess multiple lineage potentials^51^. On the other hand, it has been reported that the fraction of STRO-1 positive hMSCs significantly decreased down to 25% when the cells were cultured on plastic dishes for 10 d^43^. Therefore, although STRO-1 expression level does not necessarily coincide with the level of multipotency, it is remarkable that about 90% of the hMSCs exhibited positive signals for STRO-1 at *t* =  = 20 d both on stiff and soft substrates (Fig. 3b1). In fact, the percentages of STRO-1+ cells found on gel substrates were far beyond of the corresponding level on plastic dishes (15–20%, SI Fig. S6b). In the next step, we evaluated the functional parameters of multipotency of the hMSCs on copolymer gel substrates by biochemically inducing adipogenesis and osteogenesis at *t* = 20 d (Fig. 3). After hMSCs were cultured in induction media for 28 d, we found that hMSCs could undergo both adipogenesis (Fig. 3b2) and osteogenesis (Fig. 3b3). The apparently weaker ORO signals (for adipogenesis) could be attributed to the use of HEPES buffer necessary for pH modulation as well as to the low surface density of cells (10^4 ^cells/cm^2^) necessary for the identification of single cell response. The maintenance of multiple lineage potentials over 20 d is in striking contrast to previous studies, which showed that hMSCs were committed to specific lineages after incubation for 14 d on cross-linked polyacrylamide gel substrates with fixed elasticity^15^,^17^. Previously, Winer *et al*.^17^ suggested that hMSCs remain “quiescent” on very soft polyacrylamide gels (*E* = 250 Pa) functionalized by covalent immobilization of collagen type I and fibronectin. They demonstrated adipogenesis on soft gels, but osteogenesis could only be induced after hMSCs were transferred onto a glass substrate which has about 7 orders of magnitude larger Young’s modulus. The maintenance of multiple lineage potentials of hMSCs on PDPA-PMPC-PDPA gels over 20 d could be attributed to a decrease in the interference with the MSC lineage by avoiding the covalent immobilization of collagen type I or vitronectin, which tend to interfere with the lineage towards osteogenesis^39^,^40^,^45^.

As presented in Fig. 4, the fraction of Type C hMSCs showing positive adipogenetic ORO signals was very similar to that of Type A hMSCs, and the levels in Type B and Type D hMSCs were also comparable. The fact that the efficiency of adipogenesis does not depend on the original substrate elasticity at *t* = 0 d suggests that hMSCs would lose the mechanical memory of their contact substrates once they are cultured on either stiff or soft substrates for 10 d. The plot of circularity *γ* vs. aspect ratio *AR* (Fig. 5) enables one to discriminate the morphological phenotypes of Type A and Type B hMSCs. As presented in Fig. 5, the about 70% of Type C hMSCs can be categorized in the pattern of Type A hMSCs, while more than 90% of Type D hMScs are in the pattern of Type B, respectively. Moreover, it has been demonstrated that the nematic order parameter of actin stress fibers exhibited the same tendency (SI Fig. S2) as the morphological phenotypes. From the results presented in Fig. 4, SI Fig. S2, and Fig. 5, we therefore concluded that 10 d would be sufficient for hMSCs to adopt their morphology and cytoskeletal order, which finally determines their lineage commitment. Maintenance of multiple lineage potentials on PDPA-PMPC-PDPA copolymer gels found both on stiff and soft substrates clearly indicates that the substrate elasticity alone may not be sufficient to determine whether hMSCs retain multiple lineage potential or undergo terminal differentiation.

To further investigate the characteristic response time for hMSCs to adopt their morphology to an abrupt change in substrate elasticity, we have plotted the change in aspect ratio *AR* as a function of time (Fig. 6). From the change in intensity of the principal peaks with the smallest *AR*, the characteristic time constants of *τ* = 3–4 d could be obtained for both Type C (soft to stiff) and Type D (stiff to soft). Recently, Yang *et al*.^30^ utilized poly(ethylene glycol)-based gels with a photodegradable cross-linker, and examined the effect of substrate softening on the expression of transcription factors YAP/TAZ in hMSCs. When hMSCs were seeded on stiff gel substrates (*E* ~ 10 kPa), these transcription factors were localized in the cell nucleus already within 1 d. Upon softening of substrates by UV irradiation (*E* ~ 2 kPa), the location of YAP/TAZ was shifted to the cytosol of hMSCs. Interestingly, such changes in transcription disappeared if hMSCs were cultured on stiff substrates for more than 7 d. Although they could change the substrate elasticity only in one direction (stiff to soft), the observed plasticity in the expression of transcription factors seemed to agree with our experimental finding: the level of adipogenetic lineage does not depend on the initial substrate elasticity at *t* = 0 d, but on the elasticity of last 10 d (Fig. 4).

Finally, we investigated whether the proliferation of hMSCs could be influenced by applying periodic mechanical stresses. As presented in Fig. 7, an increase in the frequency *f* of mechanical stress, i.e. a decrease in the duration of a mechanical step *f*^−1^, led to a monotonic decrease in the fraction of proliferating stem cells, labeled with anti-BrdU (Fig. 7). In fact, periodic mechanical stresses at high frequency (*f*^−1^ = 2 d) significantly suppressed the proliferation: only 15 ± 9% of hMSCs proliferated even after 20 d. This is distinctly different from the results from Types A and B hMSCs, where the fraction of BrdU positive cells was 25 ± 3.7%. The fitting of proliferation levels with an empirical Hill’s equation (Equation 1) yields the characteristic duration of a mechanical step causing the suppression of hMSC proliferation, *f*^−1^ = 6.5 d. Although the readouts are different, this is comparable to the critical time windows for the plasticity of adipogenesis vs. osteogenesis reported by the UV-induced stiffening (8 d)^50^ and the location of YAP/TAZ expression by the UV-induced softening (7 d)^30^. Though there have been several studies that utilize the expansion and contraction of silicon rubber substrates for osteogenic lineage^52^,^53^, this is the first quantitative demonstration of the existence of a characteristic frequency of elasticity changes beyond which hMSCs suppress their proliferation.

The use of biocompatible, stimulus-responsive synthetic copolymer gels as *in vitro* surrogate substrates has enabled us to apply periodic mechanical stresses at high frequencies, which eventually push hMSCs into a non-proliferating state without losing their multiple lineage potentials. Note that the adhesion of hMSC to PDPA-PMPC-PDPA gel substrates was solely mediated through non-covalently anchored serum fibronectin and the switching of substrate elasticity was triggered by pH modulation that does not interfere with the cell viability. Since the copolymer substrates have no reactive unhydride side chains or covalently coupled adhesion motifs (e.g. collagen type I or RGD motifs) and do not require UV irradiation that might damage stem cells, the *in vitro* niche model based on PDPA-PMPC-PDPA copolymers can provide hMSCs with not only a dynamic but also a biologically safe micro-environment for expansion: Our findings have the potential to solve some fundamental problems in clinical applications of cultured stem and progenitor cells. Expansion of stem cells such as MSCs upon long-term culture *in vitro* is confounded by ill-defined factors that invariably induce a profound impairment on the functional integrity of the cell products, as evidenced by replicative senescence, and loss of differentiation potentials after incubation on plastic dishes^42^,^54^,^55^,^56^. The ablitiy to suppress the proliferation of hMSCs without losing their multiple lineage potential might enable us to synchronize differentiation of stem cells to a specific target lineage by providing a specific biochemical induction cue at a defined time point.

## Methods

### Preparation of Surrogate Substrates

The PDPA_50_-PMPC_250_-PDPA_50_ triblock copolymer (M_n_ = 60,500; M_w_/M_n_ = 1.43) was synthesized by atom transfer radical polymerization (ATRP) as reported previously^36^,^37^. Glass cover slides were cleaned according to a modified RCA method^32^ and glued into a cell culture dish (Ø 35 mm). After spin-coating and annealing of substrates with a methanolic solution of PDPA_50_-PMPC_250_-PDPA_50_, residual methanol was removed by immersing in the culture medium (DMEM low glucose, HEPES buffered, 10% FCS). The elastic modulus of PDPA_50_-PMPC_250_-PDPA_50_ copolymer gels measured as a function of pH was presented in our previous report^32^.

### hMSCs on Stimulus-Responsive Hydrogels

Human mesenchymal stem cells (hMSCs) were isolated and cultured as described before^57^. Briefly, bone marrow from healthy donors for allogeneic transplantation was taken after written consent using guidelines approved by the Ehtic Committee on the Use of Human Subjects at the University of Heidelberg. The mononuclear cell fraction was isolated by density gradient centrifugation and seeded in plastic culture flasks at a density of 100,000 (MNC)/cm^2^ in MSCGM^TM^ (Mesenchymal Stem Cell Growth Medium, Lonza, Basel, Switzerland). After 10–14 days evolving colonies were separated and MSC further expanded. Cells of early passages were seeded on copolymer gels. Since the proliferation rate of hMSCs was very low on hydrogel substrates (Fig. 7), a seeding density of 10^4 ^cell/cm^2^ was used to monitor the morphological response of single cells. To modulate pH, we used a culture medium of DMEM low glucose medium (Sigma-Aldrich, Schnelldorf, Germany) buffered with HEPES, supplemented with 10% FCS (PAA-GE-Healthcare, Munich, Germany), 1% L-glutamine and penicillin/streptomycin (100 U/ml) (Sigma-Aldrich, Schnelldorf, Germany), and adjusted pH using either 1 M NaOH aqueous solution or 1 M HCl, respectively. Cells first adhered on stiff gels (*E* = 40 kPa), and the substrate elasticity was adjusted according to the experimental flow presented in Fig. 3a. Throughout the study, this time point was defined as *t* = 0 d. hMSCs were cultured at 37 °C in a humidified atmosphere and the culture medium was exchanged every second day.

### Immunocytochemistry

STRO-1 was labeled with FITC-conjugated anti-STRO-1 (BioLegend) for 1 h at room temperature. Actin filaments were stained with Alexa Fluor 488 Phalloidin (Invitrogen), and cell nuclei were stained with DAPI (Sigma). For osteogenic induction, hMSCs were cultured in osteogenic induction medium, supplemented with dexamethason (100 nM), L-ascorbic acid-2-PO_4_ (200 μM), and β-glycerophosphate. The adipogenic induction medium was supplemented with dexamethason (1.0 mM), 1-methyl-3-isobutylxanthine (0.50 mM), and insulin (10 mg/L). The differentiated hMSCs were stained with alizarin red S for osteogenesis and oil red O for adipogenesis^56^,^58^. For quantification of adipogenic cells the stained cells with oil red and total cell numbers were counted^59^. To determine the fraction of proliferating hMSC, the cells were incubated with BrdU reagent overnight. After cell fixation with 4% (w/v) paraformaldehyde, cells were permeabilized with 0.5% Triton X100 and incubated with Cy5-conjugated anti-BrdU antibody for 1 h, while cell nuclei were stained with DAPI. Images of DAPI and Cy5-BrdU channels were overlaid and the fraction of BrdU-positive cells was calculated manually.

### Calculation of Order Parameters of Cytoskeletons

The pixel orientational map where each orientational angle is coded with different colors was obtained by image analysis with a series of elongated Laplace functions of Gaussian filters^18^,^31^,^41^. Order parameters were determined from the histogram of the pixel numbers multiplied by the corresponding pixel fluorescence intensities at each orientation, which reflects the amounts of actin fibers at each orientation.

### Shape Analysis

Morphological response of MSCs to mechanical stimuli was monitored using an inverted microscope (TE-2000U, Nikon, Düsseldorf, Germany). Cell images were recorded with a CCD camera (A602f, Basler, Ahrensburg, Germany) using 4 × air objective with phase L (N.A. 0.13, Nikon). Shape analysis of MSCs, aspect ratio (ratio between major and minor axis) and circularity (4πA_projected_)/(L_perimeter_)^2^) determination was done using ImageJ software.

### Statistics

Each data point corresponds to means ± standard deviations from more than five independent experiments. The number of cells taken for the data analysis is stated in the figure captions. The statistical significance was evaluated by the Wilcoxon test, which is indicated in the figures.

## Additional Information

**How to cite this article**: Frank, V. *et al*. Frequent mechanical stress suppresses proliferation of mesenchymal stem cells from human bone marrow without loss of multipotency. *Sci. Rep*. **6**, 24264; doi: 10.1038/srep24264 (2016).

## Supplementary Material

Supplementary Information

## Figures and Tables

**Figure 1:**
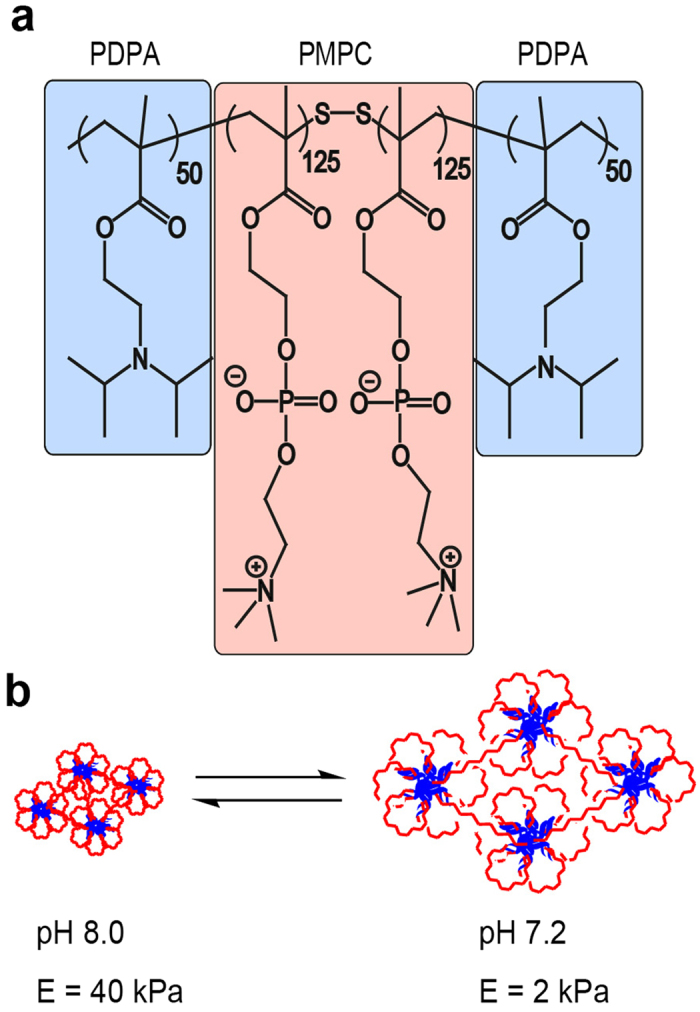
Schematic illustration of stimulus responsive substrates. Chemical structure of the pH sensitive triblock copolymer (PDPA-PMPC-PDPA) and the changes within the micellar structure induced by the pH change. pH titration between 7.2 and 8.0 enables one to reversibly switch the elastic modulus by a factor of 20.
