# Supplementary material for: Frequent mechanical stress suppresses proliferation of mesenchymal stem cells from human bone marrow without loss of multipotency
Source: Sci Rep. 2016 Apr 15;6:24264. doi: 10.1038/srep24264 (PMC4832181; doi:10.1038/srep24264)
Supplement: Supplementary Information [file srep24264-s1.pdf]

Frequent mechanical stress suppresses proliferation of mesenchymal stem cells from human bone marrow without loss of multipotency

Viktoria Frank[1,‡], Stefan Kaufmann[1,‡], Rebecca Wright[1], Patrick Horn[2], Hiroshi Y. Yoshikawa[1,3], Patrick Wuchter[2], Jeppe Madsen[5], Andrew L. Lewis[6], Steven P. Armes[5], Anthony D. Ho[2,*], and Motomu Tanaka[1,4,*]

Supplementary Information

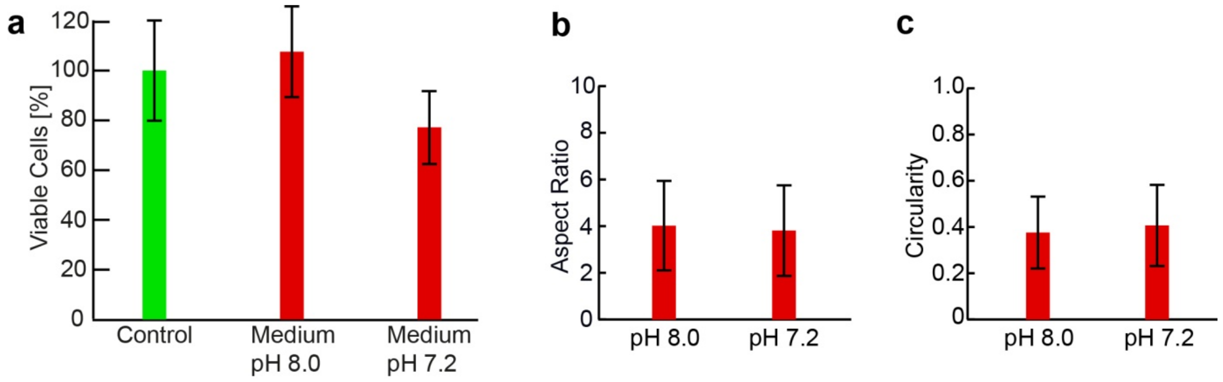

Supplementary Figure S1: Influence of pH on hMSCs on plastic dishes (control). (a) Cell viability was quantified using the WST-1 based colorimetric assay; (b) aspect ratio and (c) circularity of hMSCs on plastic dishes at t = 10 d. Data points represent mean values ± SD for n > 30 cells.

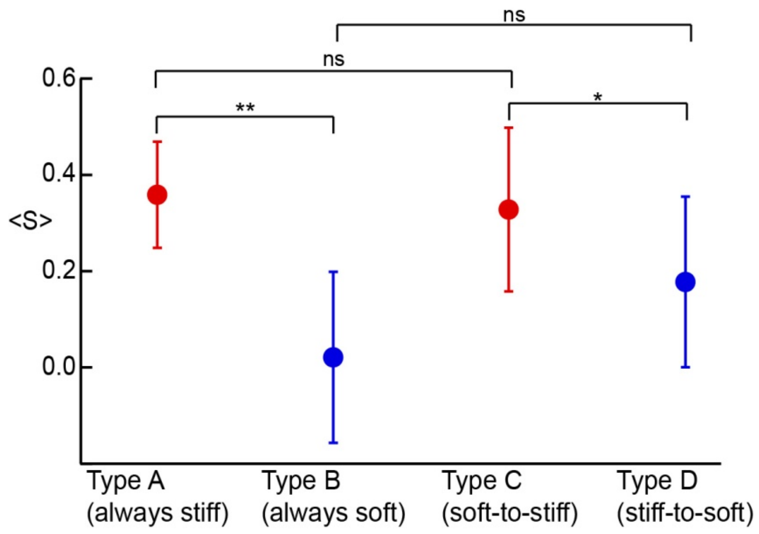

Supplementary Figure S2: Statistics of order parameters calculated for Types A - D at *t* = 20 d. Data points represent mean values ± SD for *n* > 10 cells.

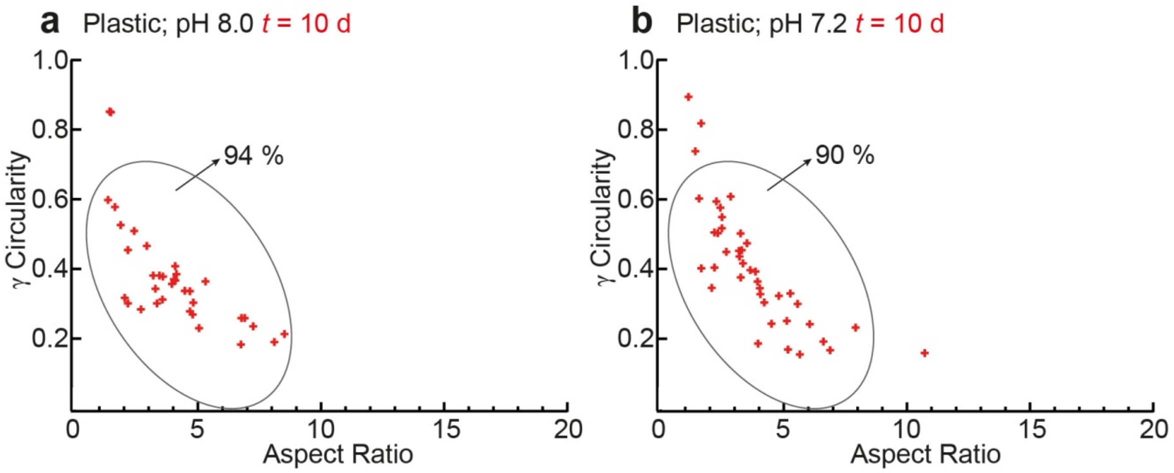

Supplementary Figure S3: Influence of pH on morphology of hMSCs on plastic dishes (control). (a) MSCs cultured on a plastic dish at pH 8.0 at *t* = 10 d, (b) MSCs cultured on a plastic dish at pH 7.2 at *t* = 10 d. *n* > 30 cells

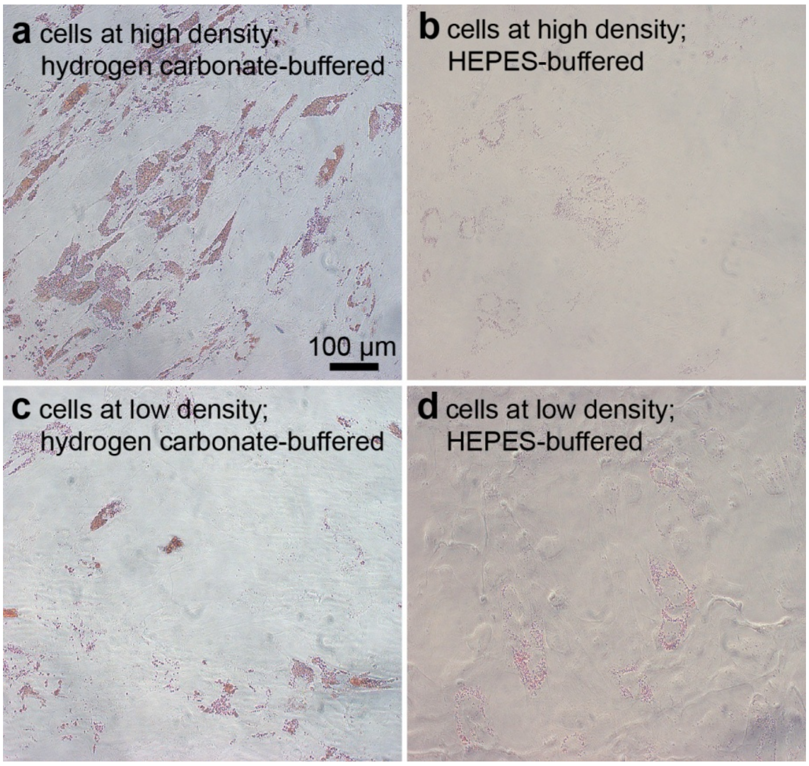

Supplementary Figure S4: Influence of medium on hMSCs adipogenesis identified by ORO staining, (control). (a) hMSCs cultured in carbonate-buffered medium at a high density (close to confluence), (b) hMSCs cultured in carbonate-buffered medium at a low density, (c) hMSCs cultured in HEPES-buffered medium at a high density (close to confluence) (d) hMSCs cultured in HEPES-buffered medium at a low density.

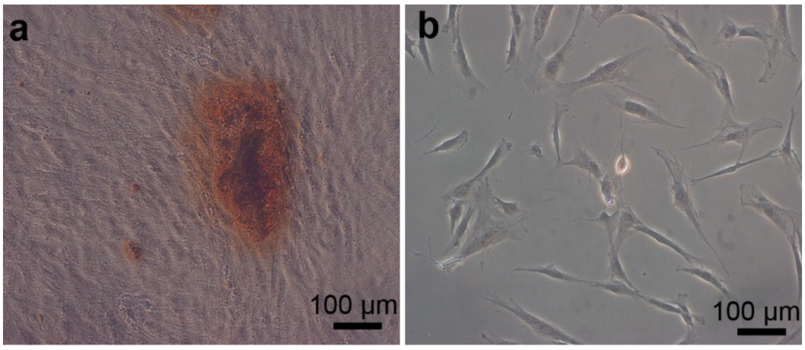

Supplementary Figure S5: hMSCs osteogenesis (control).(a) hMSCs on plastic dishes cultured in osteogenic induction medium for 21 days and stained with Alizarin red S (positive control), (b) hMSCs cultured in growth medium and stained for Alizarin red S (negative control).

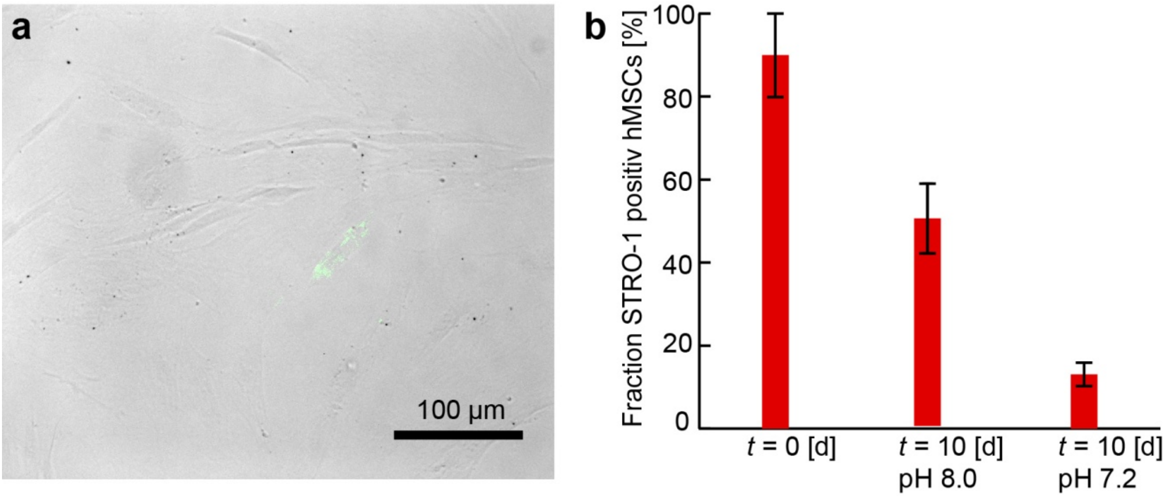

Supplementary Figure S6: hMSCs cultured on a plastic dish stained with anti-STRO-1 at t = 10 d. (a) Overlay of bright field image and fluorescence image of anti STRO-1 stain, (b) Fraction of STRO-1 positive hMSCs on plastic dishes at t = 0 d and 10 d, data points represent mean values ± SD for $n > 30$ cells.

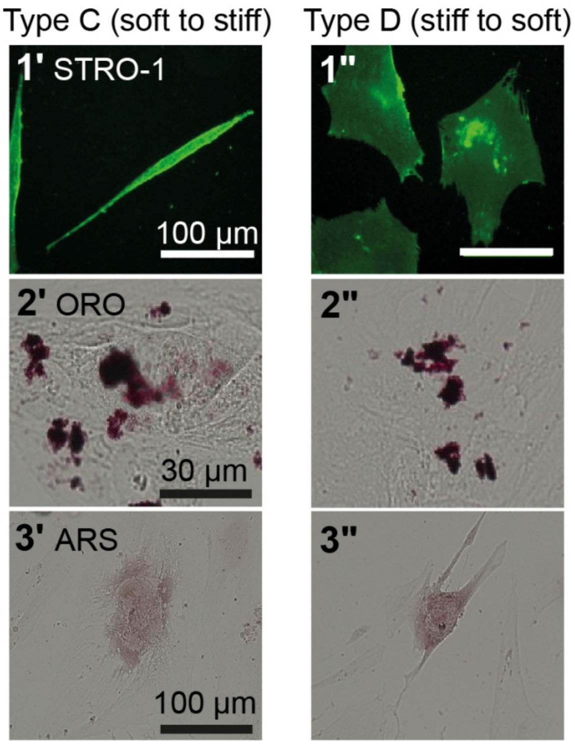

Supplementary Figure S7: hMSCs sustains multipotency after 20 d independent from substrate stiffness. Fluorescence images of hMSCs experiencing a substrate elasticity change from soft to stiff (Type C) hMSCs experiencing a substrate elasticity change from stiff to soft (Type D). Labels: (1) anti-STRO-1, (2) Oil Red O (ORO), and (3) Alizarin Red S (ARS).
